# Effects of replacement of para-grass with oil palm compounds on body weight, food intake, nutrient digestibility, rumen functions and blood parameters in goats

**DOI:** 10.5713/ajas.19.0069

**Published:** 2019-08-23

**Authors:** C. Buranakarl, S. Thammacharoen, S. Semsirmboon, S. Sutayatram, S. Chanpongsang, N. Chaiyabutr, K. Katoh

**Affiliations:** 1 Department of Physiology, Faculty of Veterinary Science, Chulalongkorn University, Pathumwan, Bangkok, 10330, Thailand; 2 Department of Animal Husbandry, Faculty of Veterinary Science, Chulalongkorn University, Pathumwan, Bangkok, 10330, Thailand; 3 Division of Functional and Developmental Science of Livestock Production, Graduate School of Agriculture, Tohoku University, Sendai 981-0845, Japan

**Keywords:** Goat, Nutrient Digestibility, Oil Palm Frond, Oil Palm Meal, Ruminal Fermentation

## Abstract

**Objective:**

The aim of the present study was to investigate the beneficial effects of dietary supplementation with oil palm frond (leaf) (OPF) with and without oil palm meal (OPM) on nutrient intake and digestibility, ruminal fermentation and growth performance in goats.

**Methods:**

Six female crossbred goats were fed for 28 days of 3 diet treatments; 100% para-grass (T1); 50% para-grass + 50% OPF (T2), and 30% para-grass + 50% OPF + 20% OPM (T3). Body weight, rectal temperature, respiratory rate, and urine volume, food intake, dry matter intake and water intake were measured daily. Nutrient digestibility was determined from five consecutive days of last week in each diet. Ruminal fluid, urine and blood were collected at the end for determination of rumen protozoa and volatile fatty acid contents, urinary allantoin excretion, blood cell count and chemistry profiles.

**Results:**

Goats fed T2 and T3 showed higher dry matter and nutrients intakes while protein digestibility was suppressed compared with those for T1. Crude fat digestibility declined in T2 but maintained after adding the OPM (T3). High fat intake by giving OPF and OPM corresponded to a higher ruminal acetate/propionate ratio (C2/C3) and serum cholesterol level. An increased urinary allantoin/creatinine ratio was found in T2 and T3 compared with T1, implying an increased number of ruminal microbes.

**Conclusion:**

Increased dry matter intake in T2 and T3 suggested that oil palm by-products are partly useful as a replacement for para-grass in goats. Replacement with the by-products increased plasma cholesterol level, which suggested that these products are a useful energy source. Changes in rumen parameters suggested an increased microbial number and activity suitable for acetate production. However, the limited digestibility of protein implies that addition of high protein feeds may be recommended to increase body weight gain of goats.

## INTRODUCTION

Goat production plays an important role in the rural economy in many developing countries because goats produce essential food items such as milk and meat. Although there are many factors such as feed and environment that affect the growth and reproductive performance of animals, it is preferable to use agricultural and natural resources specific in those regions of the world. Naturally, para-grass (*Brachiaria mutica*) is easily grown in Thailand and has been used by the farmer as a basic feedstuff for goats. However, other local plants such as white popinac (*Leucaena leucocephala*) have also been added to avoid para-grass shortage or improve palatability. In South East Asia, where oil palm tree planting is sometimes the major industry, oil palm frond (OPF) and its by-products after oil extraction may be an interesting resource as feed for animals.

The OPF as well as palm tree by-products (palm oil; PO, decanter; DC, palm kernel cakes; PKC and oil palm meal; OPM) have been extensively studied as a feed for ruminants. A study in goats showed that OPF or Napier grass + OPF *ad libitum* increased crude protein (CP) intake when compared with Napier grass [1]. The PO supplementation had no effect on growth performance but decreased cholesterol level in Black Bengal goats [2]. Feeding of PO supplemented diets, which were composed of DC, PKC and 5% PO, showed reduced organic matter digestibility after 30 days feeding of the diet containing DC. Acid detergent fiber (ADF) and neutral detergent fiber (NDF) were higher in the diet containing PKC. Ruminal ammonia-N concentration and the total protozoa number were lower in all groups given PO by-products. The ruminal volatile fatty acid (VFA) levels were also lower in groups fed PKC and PO [3]. However, no adverse effect was reported in the same animal breed. Feeding of diets containing DC or PKC resulted in higher number of the total and cellulolytic bacteria while methanogenic archaea was lower in goats fed PKC and PO [4]. Goats fed with control and PO diets also had higher daily body weight gain, slaughter and hot and cold carcass weights, compared with those fed DC and PKC diets [5]. These data suggested the possibility that OPF or palm by-products affect rumen fermentation, digestibility and growth performance of goats.

In the southern region of Thailand, palm trees have been extensively grown to make PO for biodiesel fuel under the Chaipattana Foundation. Thus, products including palm leaf and by-products of palm fruits after oil extraction known as OPM that contained kernel, mesocarp, endocarp and palm shell have been used as feed for goats. The present study was carried out to investigate the beneficial effects of dietary supplementation with OPF with and without OPM on nutrient intake and digestibility, ruminal fermentation and growth performance in goats. The findings of this study could provide more information on the benefits of these products to improve nutritional management in goat farming.

## MATERIALS AND METHODS

### Animal experiment

The protocol of this experiment was carried out in according to the institutional guidelines and was approved by Animals Care and Use Committee, Faculty of Veterinary Science, Chulalongkorn University (Animal Use Protocol No 1731032). Six healthy Black Bengal crossbred mature female goats with average body weight of 15.08±0.98 kg and age between 8 to 9 month old that belonged to Chaipattana Foundation raised at Phetchaburi province were used. All animals were kept in individual metabolic cages (2×1 m. shaped pens with an opened top and plastic floor) under a shaded barn for the entire studying period. The goats were also able to see and communicate with other goats for socialization. The animals were randomly allocated into 3 groups (2 animals in each group) and were assigned as two replications of 3×3 Latin-square designs. After 1 week of cage acclamation, each group of animals was randomly and rotationally assigned to three different diets. The diets were as follow: 100% of para-grass (treatment 1; T1), 50% of para-grass + 50% OPF (treatment 2; T2) and 30% of para-grass + 50% OPF + 20% OPM (treatment 3; T3). The compositions of the para-grass, OPF and OPM are shown in Table 1.

The para-grass (*Brachiaria Mutica*) was grown naturally without fertilizer and was cut daily in the morning (09:00 to 10:00). The cutting length was around 30 cm long. The OPF was obtained from palm trees grown inside the foundation mainly for biodiesel production. The whole leaf was cut and only the leaflet was chopped into pieces of approximate 2.5 cm long before feeding to the goats. The OPM was obtained from the PO fruit after oil extraction. In brief, the fruit moisture was removed by boiling with PO and then the fruit was compressed in order to extract the PO. The residue after oil extraction was sieved to remove the big pieces of shell and the remaining was used as OPM to feed the goats.

The diets were offered to the animals *ad libitum* twice daily at 07:00 h and 18:00 h with free access of water. Each experimental period for each diet lasted for 4 weeks. After finishing one diet period, the animals were subjected to another diet for further 4 weeks, which was repeated until all 3 diet experiments were complete.

### Sampling and parameter measurement

Body weight was measured once a week before morning meal. The daily rectal temperature (RT) and respiratory rate (RR), which were measured every day before morning meal, were averaged for last two weeks of each diet. The RT and RR were recorded using digital thermometer and stethoscope, respectively during 08:00 to 09:00 along with the collection of ambient temperature and humidity to obtain the temperature humidity index.

Daily food intake (FI), dry matter intake (DMI) and water intake (WI) were daily recorded and averaged for the entire 28 days.

In order to evaluate the nutrient digestibility, samples of both feed offered and refusals and all feces were collected daily for 5 consecutive days at the end of each treatment and the data were averaged. The feces were dried in microwave oven at 400 watts of heat intensity for 30 min and weighted for the five consecutive days during the last week of each diet experiment. Samples collected in each day were mixed thoroughly and kept frozen at −20°C for later chemical analysis.

Collection of ruminal fluid, urine and blood was performed at the end of last week of each diet experiment for determination of rumen protozoa and VFA contents, urinary allantoin excretion and blood cell count including chemistry profiles. Samples collected were kept frozen at −20°C for later chemical analysis.

Rumen fluid samples were collected using an orogastric tube connected to a syringe while the goats were under a few minutes of physical restrain by experienced personnel. Approximately 20 mL of fluid was taken 2 h after morning meal. For determination of VFA concentrations, the ruminal fluid was filtered through two layers of cheesecloth, then 2 to 3 drops of toluene were added to inhibit fermentation. Filtered samples were then frozen at −20°C for later analysis

Total urine was collected using plastic containers beneath the cage floor with 10% sulfuric acid solution (13 mL of 10% H_2_SO_4_ per urine 100 mL) added to prevent nitrogen loss. Final urine pH was kept below 3. Total urine volume was measured separately by day and night times and then combined. All urine samples were diluted with distilled water to minimize the crystallization of urinary compounds and then kept in a refrigerator.

Approximately 6 mL of blood was collected from the left jugular vein into a syringe and put into the ethylenediaminetetraacetic acid-added and heparinized tubes (3 mL each) at 09:00 on the same day as the ruminal fluid collection. The samples were kept on ice for later analysis of complete blood count and chemistry profiles.

### Analytical procedure

#### Analysis of diet compositions

All diets were dried overnight in oven at 60°C until weights were constant to determine dry matter (DM). Samples after drying and grinding were used to determine diet composition. The DM, crude fat, CP, and crude ash were determined by the method of AOAC [6] and NDF and ADF were determined by the method of Van Soest et al [7].

The chemical compositions of the OPF (DM 45.1%, ADF 46.5%, NDF 61.1%, CP 13.9%, ether extract [EE] 4.2%, and ash 12.1%) and OPM (DM 92.6%, ADF 46.0%, NDF 67.1%, CP 10.5%, EE 12.7%, and ash 6.1%) were estimated as percentage of DM.

In order to obtain the nutrient digestibility, the composition in feces was analyzed along with diets. The total digestible nutrient (TDN) was calculated according to Lofgreen [8]. The total tract nutrients digestibility was calculated by the following formula

$$\begin{array}{l} \text{Digestibility}=(\text{nutrient concentration in consumed feed}\\ -\text{nutrient concentration in feces})/\text{Nutrient concentration in feed} \end{array}$$

#### Determination of ruminal protozoa number

The protozoa counting and determination of the types were analyzed following the reported method by Dehority [9]. One ml of ruminal fluid was fixed with 10% NaCl and methylene blue solution before loaded into hemocytometer to evaluate total number and relative percentage of each type of protozoa under a light microscope × 400 (Olympus, Tokyo, Japan).

#### Determination of ruminal volatile fatty acid concentration

On the day of measurement, a 5 mL of rumen fluid was thawed, and was mixed with 1 mL of 25% of meta-phosphoric acid and formic acid (3:1) in a centrifuge tube. The mixed samples, after being allowed to precipitate for 30 min, were centrifuged at 4,000 rpm for 20 min, and the clear supernatant was collected into another tube, a part (1 μL) of which was injected into gas chromatography (GC-2010, Shimadzu, Tokyo, Japan), equipped with a 30 m×0.25 mm×0.50 μm film fused silica capillary columns (DB-WAX, Agilent, Santa Clara, CA, USA). Injector and detector temperatures were 225°C and 250°C, respectively. Carrier gas-Helium total and column flow were 35.8 and 1.56 mL/min, respectively.

#### Determination of urinary allantoin and creatinine ratio

The allantoin was analyzed by colorimetric method according to Young and Conway [10]. The urinary protein creatinine was measured by colorimetric method using automated analyzer (ILab 650, Warfen, Milano, Italy).

#### Determination of complete blood count and chemistry profiles

The complete blood cell count was determined using an automated analyzer (XT-2000i, Sysmex, Kobe, Japan). The blood chemistries including blood urea nitrogen, glucose, alanine transferase (ALT), alkaline phosphatase (ALP), total protein, albumin, cholesterol, and triglyceride were analyzed by automated analyzers (LIASYS 2, AMS Alliance, Rome, Italy).

### Statistical analysis

All data are represented as the mean and standard error of mean (n = 6). The differences among diet treatments were compared using analysis of variance based on Latin square model of SAS program. The pairwise comparisons were performed using Duncan new multiple rank test. Difference was considered significant when p-value was less than 0.05.

## RESULTS

### Diet composition

The compositions of experimental diets (T1, T2, and T3) are shown in Table 1. The DM and fat contents in oil palm compounds were higher than para-grass. As a result, the DM content in T2 and T3 feed were 2 times higher than in T1. The fat content in T2 and T3 was 1.5 and 2 times higher, respectively, than that in T1. Between three diets, TDN was slightly higher in T2 and T3 when compared with T1.

### Body weight, intakes of food, dry matter and water, rectal temperature, respiratory rate

The total heat index during the experiment was not different among treatments (78.87±0.44, 78.84±0.43, and 78.55±0.46 for T1, T2, and T3, respectively), but WI was higher in T3. The body weight and % BW change of goats were not different among three groups (Table 2).

The FI recorded for 28 days of treatment period was significantly smaller in T2 and T3 than in T1, where T3 was significantly smaller than T2 (p<0.05). The DMI was higher in T2 and T3 than in T1 (p<0.05).

The levels of RT and RR were not different among groups.

### Nutrient intakes and apparent digestibility

The average FI, DMI, and nutrient intakes during the last week (week 4) are shown in Table 3. Feed intake in T2 and T3 were significantly smaller than T1 (p<0.05), where that of T3 was the lowest. However, the intakes of DM and all nutrients were significantly higher in both T2 and T3 than in T1 (p<0.05). Protein and fat intakes were higher in T2 and T3 than T1 (p<0.05).

The nutrient digestibility measured on the last 5 days of each treatment periods are also shown in Table 3. Although no differences in the digestibility of DM, ADF, and NDF were found among three groups, the protein digestibility was significantly decreased by replacement with oil palm-related compounds (p<0.01). On the fat digestibility, T2 showed a lower value than other two groups (p<0.05).

### Ruminal protozoa number, volatile fatty acid contents and urinary allantoin excretion

The species and number of protozoa and VFA contents in the rumen fluid are shown in Table 4. Although no difference was shown in the total number of protozoa among three groups, the percentage of *Dasytricha* spp. for T3 was higher than that for T1 and T2 (p<0.05).

On ruminal VFA profiles, although there was no difference for each acid concentration among three groups, the C2/C3 ratio was significantly increased by replacement with oil palm-related compounds (p<0.01), and that was the largest in T3.

The urinary allantoin/creatinine ratio was significantly increased by replacement with oil palm-related compounds (p<0.001).

### Blood cell counts and chemistry profiles

The complete blood cell counts were not different among three groups (Table 5). Blood chemistry profiles showed significant increases in ALT in T2 and T3 and ALP in T3 (p<0.05), although these values were within normal ranges. Serum cholesterol concentration was significantly increased by replacement with oil palm-related compounds and was the largest in T3 (p<0.05).

## DISCUSSION

Since goats are unable to digest a large amount of plant cell walls because of a relatively short transit time in the rumen [11], goats prefer to eat relatively young but stiff tree leaves, which have highly digestible cellular nutrients such as proteins and carbohydrates. Therefore, goats require high quality diets.

The palm leaves, with typical stiff tree leaves, and their by-products have been widely used as feed for ruminants, especially in areas rich in palm trees planting [12]. The protein content in OPFs, normally composed of petiole, leaves and rachis, growing in Thailand is reported to be 6.2% [13]. Our data showed a higher protein level of 13.9% in the present study. This is because only oil palm leaves were used since they are richer in protein and fat contents than palm fronds [14].

The PO resources involve many kinds of by-products; i) palm kernel expeller, in which the palm kernels were ground and screwed by pressing with and without flaking and cooking, ii) PKC, which is made from palm kernel oil via solvent extraction, iii) palm oil sludge, iv) palm pressed fiber, v) OPF, vi) oil palm trunks, and vii) whole fruit after oil extraction known as OPM. The OPM has a high content of DM and crude fat.

When para-grass was replaced with palm-related by-products to make experimental diets (T2 and T3), CP contents were adjusted at a similar level (ca. 11%), which was reasonable because the daily requirement of feed protein is 11% for meat producing goats with average body weight of 80 to 120 pounds [11]. Although T3 diet had the largest crude fat content, the percentage in all diets did not exceed 5%, which is considered as an under limitation for a feed of small ruminants [11].

Our results showed reduction in feed intake when replacing with OPF in the diets with or without OPM. However, DMI was higher in T2 and T3 than T1, suggesting better palatability of the by-products than para-grass alone. Higher DMI was also reported in a previous study, in which goats were fed a combination of palm frond and Napier grass [1]. Higher palatability when replacing with PO by-products was not due to fat content since DMI was not parallel with fat content of T2 and T3. A previous study in goats, however, showed that PO supplementation failed to increase DMI [15]. Alternatively, dietary sugar content may be involved in palatability since palm trees contain more sugar rather than starch [16], or a physical feature of hard leaves being preferred by goats.

Although OPF contains high cellulose, hemicellulose and lignin [17], lignocellulose and lignin are hardly hydrolyzed due to limited enzyme activity of ruminal microorganisms [18]. However, the digestibility of ADF and NDF was not changed by replacement with OPF (T2 and T3). The results may be due to the reason that all 3 diets used were roughage-rich feeds. On this issue, previous studies showed that different palm by-products caused variable results of ADF and NDF digestibility [3], and that supplement of PO in diets did not change NDF digestibility [19]. Although it is reported that saturated fats at higher concentration (6% or more) caused the depression in NDF digestion [20], the oil concentration employed in our study may not be enough to affect the digestibility because it was 2.5% and 3.6% in T2 and T3, respectively.

Protein digestibility was reduced in the diets replaced with OPF. However, there is discrepancy with previous studies which reported a higher protein digestibility obtained by adding PO [19], and no effect by adding DC, PKC, and 5% PO to a concentrate diet [3]. The decreased protein digestibility in our study may be a result of increased passage rate through the gastrointestinal tract. Greater DMI was associated with an increased fluid passage rate [21]. In the present study, animals showed higher DMI in T2 and T3 than in T1. Moreover, goats at T3 diet had the highest WI, which may have played additional role to increase the passage rate and consequential reduction in protein digestibility.

The urine data suggest the possibility of an increased conversion of feed proteins into microbial proteins by replacement with palm-related compounds because the ratio of urinary allantoin/creatinine was significantly higher in T2 and T3. The increase in allantoin urinary excretion may be related to an increased DMI in T2 and T3. A study with calves showed that purine derivative excretion was positively related to feed intakes [22]. Our results showed that total protozoa tended to be higher, particularly *Isotricha* spp., when OPM was fed. The *Isotricha* spp. and *Dasytricha* spp. which are Holotrich ciliates can synthesize and degrade carbohydrates and sugars. In term of nitrogen metabolism, Holotrichs can both synthesize amino acids and utilize preformed amino acids released by proteolysis from ingested bacteria or plant materials [23]. Thus, changes in rumen fermentation and microorganism population may also account for low protein digestibility since the level of bacterial flow to the intestinal tract is one of the factors that affect fecal nitrogen output [24]. Nevertheless, previous studies showed variable results where palm by-products such as DC and PKC supplement showed a reduction in the number of total protozoa count but had an increased total bacteria number compared with control diet [3,4]. However, adding 5% PO in the diet could decrease bacterial population due to toxic effect of oil [4]. It is suggested that the optimal concentration of oil and the characteristic of PO products are crucial for ruminal microbial environment. Our results showed that body weight was not changed at 28 days after replacing with OPF and OPM. Thus, no negative effect on the body weight was caused by replacing with oil palm by-products as a part of roughage diet.

In addition to reduced protein digestibility, the crude fat digestibility was also reduced when OPF was fed in T2. This limited reduction digestibility in T2 may not be significant because it was not confirmed in T3, where the fat content was higher than T2. However, the lower fat digestibility may also be due to changes in rate of gastrointestinal passage or rumen environment, by a similar mechanism discussed for protein digestibility. However, fat digestibility in T3 was preserved compared with that in T2. Adding PO to diets in goats was reported to increase EE digestibility [19]. This finding was similar to that found in lambs supplement with PO [25]. Feeding high fat diet caused more ruminal triglyceride hydrolysis than a conventional diet in cattle [26]. Different chain length of fatty acids in PO, mainly C16:0 and C18:1 [27], may recover the fat digestibility and lipid metabolism. However high fat content may cause limitation of fat digestibility as reported in a study with sheep given lipid residue of biodiesel from PO [28].

The data on ruminal short-chain fatty acid measurement showed C3 tended to be lower after giving palm-related compounds, resulting in higher C2/C3 ratio. Higher C2/C3 ratio supports the data that the intake of ADF and NDF was increased as shown in Table 3, and the idea that OPF and OPM rich in fats may be a useful energy source. The result was different from that reported in steers, in which PO decreased C2/C3 ratio 2 hrs after feeding at low dosage of PO (200 g/d) [29].

Palm-related compounds when given to goats did not affect the general health as monitored by complete blood cell counts and chemistry profiles, which were in normal reference ranges. Increased ALT and ALP may be related to fat degradation and utilization. High fat content in palm-related compounds increased plasma triglyceride and cholesterol levels, which support palm by-product fat as a good energy source. The PO contained 83% of triglyceride among 84% of neutral lipids when extracted with organic solvent [30]. Similar results were found in sheep with increased plasma high density lipoprotein-cholesterol after 2 months of feeding 50% OPF plus 50% control diet [31]. However, one study found that PO could reduce serum cholesterol in Black Bengal goats 60 days after giving the oil [2]. In monogastric animals, oil palm phenolic acid with vitamin E has a high protective effect against development of arteriosclerosis lesions in rabbit model [32]. The PO is the richest natural source of vitamin E, which contained 70% tocotrienols and 30% tocopherol [33,34]. The lack of plasma-lipid-lowering-effect in the present study may be due to different cholesterol metabolism in the liver of the ruminant from monogastric animals. Although other blood chemistry profiles of goats were unchanged, increased levels of gamma glutamyl transferase, ALP, glutamic oxaloacetic transaminase, creatinine and creatine kinase were reported in cattle fed PKC for 125 days [35].

## CONCLUSION

The PO frond will be suitable as a roughage source in the areas of palm plantation since it is a low-cost by-product available throughout the year as an eco-feed. Partial replacement of para-grass with the by-products increased plasma cholesterol level, which suggested that these products are a useful energy source. Changes in rumen parameters suggested increased microbial number and activity suitable for acetate production. However, the limited digestibility of protein implies that the addition of high protein feeds may be needed to increase body weight gain of goats.

## Figures and Tables

**Table 1:** Nutritional composition of experimental diets (% DM)

Items	DM	ADF	NDF	CP	EE	Ash	TDN
Treatment 1 (paragrass)	15.6	35.4	58.4	11.4	1.7	11.9	43.7
Treatment 2 (paragrass + OPF)	30.5	36.4	52. 9	11.5	2.5	10.6	49.7
Treatment 3 (paragrass + OPF + OPM)	35.3	38.9	54.4	11.3	3.6	9.8	49.2

DM, dry matter; ADF, acid detergent fiber; NDF, neutral detergent fiber; CP, crude protein; EE, ether extract; TDN, total digestible nutrients; OPF, oil palm frond; OPM, oil palm meal.

**Table 2:** Changes of body weight, intake of feed, dry matter and water, rectal temperature and respiratory rate in goats fed three different diets recorded for a period of 28 days duration

Items	T1	T2	T3	SEM	p-value
BW (kg)	15.6	15.7	15.6	0.4	0.987
% BW change	1.39	5.04	4.89	0.80	0.311
FI (% kg BW)	11.33^a^	9.37^b^	8.62^c^	0.34	0.0001
DMI (% kg BW)	2.10^b^	2.83^a^	2.80^a^	0.06	0.0001
WI (L/d)	0.20^b^	0.23^b^	0.37^a^	0.02	0.004
RT (°C)	38.4	38.4	38.4	0.04	0.982
RR (breaths/min)	21.8	21.1	20.4	0.4	0.101

SEM, standard error of mean; BW, body weight; FI, feed intake; DMI, dry matter intake; WI, water intake; RT, rectal temperature; RR, respiratory rate.

a–c Means within a row with different superscripts differ (p<0.05).

**Table 3:** The average nutrient intakes and apparent nutrient digestibility measured for 5 days at the end of each period in goats fed three different diets

Items	T1	T2	T3	SEM	p-value
Nutrient intakes (percent of body weight/d)					
Feed	12.21^a^	10.00^b^	8.89^c^	0.41	<0.0001
DM	2.29^b^	3.03^a^	2.91^a^	0.10	0.0002
ADF	0.79^b^	1.07^a^	1.07^a^	0.04	0.0002
NDF	1.32^b^	1.55^a^	1.53^a^	0.04	0.009
Protein	0.27^c^	0.36^a^	0.34^b^	0.01	<0.0001
Fat	0.04^c^	0.08^b^	0.10^a^	0.01	<0.0001
Apparent digestibility (%)					
DM	47.9	52.3	50.5	1.8	0.408
ADF	35.7	30.0	28.3	2.6	0.126
NDF	48.3	46.2	45.8	1.9	0.674
Protein	65.3^a^	60.7^b^	55.4^c^	1.6	0.003
Fat	56.5^a^	42.7^b^	57.4^a^	2.2	0.001

SEM, standard error of mean; DM, dry matter; ADF, acid detergent fiber; NDF, neutral detergent fiber.

a–c Means within a row with different superscripts differ (p<0.05).

**Table 4:** Ruminal protozoa and volatile fatty acid contents and urinary allantoin creatinine ratio in goats fed three different diets

Items	T1	T2	T3	SEM	p-value
Rumen protozoa					
Total count (cell/mm^3^)	60.4	55.0	97.2	13.6	0.320
*Isotricha* spp. (% of total)	0.2	7.2	4.3	1.3	0.157
*Dasytricha* spp. (% of total)	1.4^b^	0^b^	5.7^a^	1.2	0.029
*Entodinium* spp. (% of total)	54.7	69.2	59.3	3.6	0.284
*Diplodiniiae* spp. (% of total)	39.3	22.1	29.4	3.3	0.159
*Epidinium* spp. (% of total)	4.4	1.6	1.4	0.9	0.408
Ruminal VFA (mmol/L)					
Acetic acid (C2)	101.8	110.2	105.5	7.8	0.774
Propionic acid (C3)	28.2	27.5	23.8	1.5	0.227
Butyric acid (C4)	6.6	7.3	6.3	0.5	0.495
C2/C3	3.61^c^	4.17^b^	4.78^a^	0.33	0.004
Rumen pH	7.23	7.33	7.10	0.05	0.075
U-allantoin/UCr (mg/mgCr)	1.19^b^	2.00^a^	1.87^a^	0.10	0.0002

SEM, standard error of mean; VFA, volatile fatty acid; U-allantoin, urinary allantion; UCr, urinary creatinine.

a–c Means within a row with different superscripts differ (p<0.05).

**Table 5:** The complete blood count and chemistry profiles in goats fed three different diets

Items	T1	T2	T3	SEM	p-value
Complete blood count					
PCV (%)	36.0	35.0	36.8	0.8	0.102
HGB (g/L)	112.5	110.0	114.7	2.3	0.210
WBC (×10^9^/L)	20.8	22.9	20.8	0.6	0.128
Platelets (×10^9^/L)	505.3	522.5	524.0	29.1	0.969
Blood chemistry					
Blood glucose (mmol/L)	3.86	3.73	3.92	0.09	0.550
BUN (mmol/L)	5.57	5.38	5.22	0.17	0.676
Creatinine (μmol/L)	113.0	113.5	114.2	2.0	0.970
ALT (IU/L)	15.0^b^	19.6^a^	21.8^a^	1.5	0.003
ALP (U/L)	238^b^	258^b^	350^a^	65	0.033
Protein (Biuret) (g/L)	56.7	52.5	53.3	1.4	0.610
Protein (ref) g/L)	68.7	67.3	69.0	1.0	0.509
Albumin (g/L)	38.0	36.0	38.3	1.5	0.092
Cholesterol (mmol/L)	2.44^b^	2.55^ab^	2.92^a^	0.16	0.047
Triglyceride (mmol/L)	0.31	0.38	0.47	0.03	0.127

SEM, standard error of mean; PCV, packed cell volume; HGB, hemoglobin; WBC, white blood cell; BUN, blood urea nitrogen; ALT, alanine transminase; ALP, alkaline phosphatase; ref, refractometer.

a,b Means within a row with different superscripts differ (p<0.05).
